# Opioid−free anesthesia attenuates perioperative immunosuppression by regulating macrophages polarization in gastric cancer patients treated with neoadjuvant PD-1 inhibitor

**DOI:** 10.3389/fimmu.2024.1438859

**Published:** 2024-09-23

**Authors:** Wenjian Liu, Chaopeng Ou, Ruifeng Xue, Xiaohua Yang, Yaqi Ye, Xudong Wang, Jingdun Xie

**Affiliations:** ^1^ Department of Anesthesiology, State Key Laboratory of Oncology in South China, Guangdong Provincial Clinical Research Center for Cancer, Sun Yat-sen University Cancer Center, Guangzhou, Guangdong, China; ^2^ Department of Anatomy and Neurobiology, Zhongshan School of Medicine, Sun Yat-sen University, Guangzhou, Guangdong, China

**Keywords:** opioid-based anesthesia, opioid-free anesthesia, macrophages polarization, locally advanced gastric cancer, PD-1 inhibitor

## Abstract

**Background:**

Opioid anesthesia can modulate the impaired immune response and opioid-sparing anesthesia may preserve immune functions. This study was performed to assess the effects of opioid-free anesthesia (OFA) and opioid-based anesthesia (OA) on perioperative macrophages differentiation, cytokine changes, and perioperative complications in locally advanced GC (LAGC) patients.

**Methods:**

We used quality of recovery-15 (QoR-15) questionnaire scores and visual analog scale (VAS) scores to compare postoperative quality of recovery and pain level. In addition, the adverse reactions of patients in the two groups were compared. The perioperative serum level of inflammatory cytokines and the ratio of macrophage subtypes were detected.

**Results:**

The OFA group had significantly longer extubation time and PACU stay, whereas the OA group had significantly higher rate of hypotension, higher doses of norepinephrine, higher PONV and dizziness rate, and delayed flatus passage time. The QoR-15 score on postoperative 24 h was significantly higher in OFA group than in OA group. At the end of or after the surgery, the OFA group had higher levels of interleukin (IL)-12, IL-1β, tumor necrosis factor (TNF)-α, CD68+CD163− macrophage rate, but lower levels of IL-10, transforming growth factor (TGF)-β, and CD68+CD163+ macrophage rate, indicating OFA attenuated perioperative immunosuppression by diminishing M2 and promoting M1 macrophage polarization. And the reversal tendency is more obvious in LAGC patients with neoadjuvant PD-1 inhibitor.

**Conclusions:**

The OFA may attenuate perioperative immunosuppression by diminishing M2 and promoting M1 macrophage polarization in LAGC patients with neoadjuvant PD-1 inhibitor.

**Clinical trial registration:**

http://gcpgl.sysucc.org.cn, identifier 2022-FXY-001.

## Introduction

Gastric cancer (GC) ranks as the fifth most prevalent malignancy and the fourth most lethal neoplasm globally, with scarce therapeutic alternatives and dismal patient prognosis. It is noteworthy that locally advanced GC (LAGC) constitutes the bulk of newly detected gastric malignancies annually (1, 2). Currently, the principal therapeutic options for LAGC comprise surgical resection, chemotherapy, and immunotherapy (3–5). Immune checkpoint inhibitors (ICIs) targeting programmed cell death 1 (PD-1) can increase pathological response rate (pCR) (6–8) and improve overall survival (OS) in GC (9–11). Efficacy of PD-1 inhibitor can be influenced by tumor microenvironment (TME), while immunosuppressive TME correlates with poor prognosis and resistants to chemotherapy (12–14) and PD-1 inhibitor (15, 16). Evidence shows anesthesia may induce immunosuppression in patients during the perioperative period, thus adjusting anesthetic schemes to attenuate perioperative immunosuppression may be beneficial to GC patients.

Opioids are the most potent analgesics in anesthetic regimens to induce perioperative analgesia, sedation and suppression of sympathetic nervous system (17). The adverse effects related to opioid administration encompass excessive analgesia (18), persistent postoperative pain (18), respiratory depression (19), postoperative nausea and vomiting (PONV) (20) and postoperative delirium (21). More importantly, considerable evidences showed that endogenous and exogenous opioids regulate immune function by changing biochemical pathways and proliferative characteristics of cell components of immune system (22, 23). Previous research have indicated that opioid-sparing anesthesia may preserve immune functions in esophageal cancer (24) and breast cancer (25).

Macrophages infiltrate the TME majority in neoplasms and exert a crucial role in tumor immunity. During oncogenesis, the tumor associated monocytes/macrophages (TAMMs) in circulating blood are chemoattracted into tumor focus and differentiate into tumor associated macrophages (TAMs) (26, 27). Macrophages exhibit plasticity under various kinds of stimuli and can be polarized into distinct functional phenotypes, classical activation (M1, marked as CD68+CD163−) or alternative activation (M2, marked as CD68+CD163+) (28–30). M1 phenotype can eliminate invading microbes and cancer cells by producing pro-inflammatory cytokines, such as interleukin (IL)-12, IL-1β, and tumor necrosis factor-α (TNF-α) (28–30). On the other hand, M2 phenotype can foster immunosuppression and immune escape in neoplasms progression by secreting anti-inflammatory cytokines, such as IL-10 and transforming growth factor-β (TGF-β) (28–30). In GC, the presence and density of M2 macrophages correlate with poor prognosis and resistant to chemotherapy (12–14, 31) and PD-1 inhibitor (15, 16).

Nevertheless, scant studies have elucidated the influence of opioids on the TME of GC patients, especially in LAGC patients with neoadjuvant PD-1 inhibitor. Hence, this research aimed to compare the opioid-free anesthesia (OFA) and opioid-based anesthesia (OA) on perioperative macrophages differentiation, cytokine alterations, and perioperative complications of LAGC patients.

## Methods

### Patients selection and exclusion

The single-center, prospective and randomized trial was approved by the Ethics Committee of Sun Yat-Sen University Cancer Center (SYSUCC) (SL-B2022-299-02), and registered in the SYSUCC Clinical Trial Register (http://gcpgl.sysucc.org.cn, identifier: 2022-FXY-001).After obtaining the written consent from GC participants, we enrolled LAGC patients aged 18–60 years with American Society of Anesthesiologists (ASA) I-III who had laparoscopic radical resection of LAGC after neoadjuvant therapy in SYSUCC from July 2022 to December 2023. We excluded patients who were: (1) pregnant, (2) allergic to any experimental drug or its ingredients, (3) suffering from central nervous system disorders (such as epilepsy, cerebral infarction, or cerebral hemorrhage history), (4) having a history of chronic pain, alcohol, or drug abuse, (5) converted to open surgery, (6) transfused perioperatively, or (7) having severe cardiac, pulmonary, hepatic, renal, or endocrine diseases. The eligible patient gave their written informed consent after agreeing to participate.

### Sample size calculation and masking method

We measured the inflammatory response, the main outcome of the study, by the level of IL-10. Previous research on IL-10 showed that its *in vivo* standard deviation (SD) is around 3.8 pg/ml. We calculated that we needed twenty patients to detect a decrease of 3.4 pg/ml deviation with an α-value of 0.05 and a power of 0.8. We enrolled twenty-five patients to account for potential dropouts. Moreover, we divided LAGC patients based on the type of neoadjuvant therapy (chemotherapy or PD-1 inhibitor plus chemotherapy), and we doubled the sample size accordingly.

At the beginning of the study, patients were randomly assigned to two groups (group OA and group OFA) using computer-generated codes. The doctors who performed postoperative evaluation and biological testing did not know the group assignments until the end of the follow-up and the final analysis.

### Anesthetic management

All patients routinely fasted for 8 h and abstained from drinking for 2 h before surgery. After entering the operating room, electrocardiography (ECG), pulse oximetry, invasive blood pressure, and temperature measurements were routinely monitored. The IV bolus medications ready to use were: Atropine 0.1 mg/mL and Norepinephrine 40 mcg/mL. Pre-oxygenation was administered for 2 min before anesthesia induction. The anesthetic management administration regimen differed between the groups (**Table 1**). Anesthetic depth was monitored using the bispectral index sensor (BIS), and the sevoflurane concentration and anesthetic drug doses (remifentanil or esketamine) were adjusted to keep BIS values between 45 and 60 during the anesthesia maintenance. Hypotension was defined as a decrease in mean blood pressure [MBP] > 30% of baseline or MBP < 65 mmHg, and Hypertension was defined as an increase in MBP > 30% of baseline or MBP > 90 mmHg. Norepinephrine was administrated as a continuous infusion at the discretion of the attending physician to keep MBP within the target blood pressure (within ±20% of the baseline MAP). Bradycardia (defined as HR < 50 beats/min) will be treated with intravenous atropine 0.3–0.5 mg. Tachycardia (defined as HR > 100 beats/min) will be treated with intravenous esmolol 20 mg.

**Table 1:** Anesthetic management protocol.

Opioid-Based Anesthesia Induction	Opioid-Free Anesthesia Induction
Anesthesia induction	
Anxiolytic agent: 1 min prior to induction Midazolam 2–3 mg IV	Anxiolytic agent: 1 min prior to induction Midazolam 2–3 mg IV
Sufentanil 0.2-0.4mcg/kg IV	Dexmedetomidine 1 mcg/kg IV in 10 min
	Lidocaine 0.2mg/kg IV
	Esketamine 0.2-0.4 mg/kg IV
Propofol 2–3 mg/kg IV	Propofol 2–3 mg/kg IV
Cisatracurium 0.2mg/kg IV	Cisatracurium 0.2 mg/kg IV
Nausea and vomiting management	Nausea and vomiting management
Regional anesthesia: US-guided bilateral transversus abdominis plane and rectus sheath blocks (20 mL 0.25% ropivacaine in each block)	Regional anesthesia: US-guided bilateral transversus abdominis plane and rectus sheath blocks (20 mL 0.25% ropivacaine in each block)
Anesthesia maintenance	
Cisatracurium 0.2 mg/kg/h on continuous infusion	Cisatracurium 0.2 mg/kg/h on continuous infusion
Sevoflurane with a MAC (Minimal alveolar concentration) of 1-1.2	Sevoflurane with a MAC (Minimal alveolar concentration) of 1-1.2
Remifentanil 0.1-0.2 mcg/kg/min on continuous infusion	Esketamine 0.2-0.4 mg/kg/h on continuous infusion (stopped 30 min before end of surgery)
	Dexmedetomidine 0.5 mcg/kg/h on continuous infusion (stopped 30 min before end of surgery)
Initial loading dose: Sufentanil 0.15 mcg/kg + Flurbiprofen 50mg IV	Initial loading dose: Esketamine 0.15 mg/kg + Flurbiprofen 50mg IV
Recovery and postanesthesia pain management	
Neostigmine (up to 2 mg) + Atropine (0.2–1 mg) IV	Neostigmine (up to 2 mg) + Atropine (0.2–1 mg) IV
Patient-controlled intravenous analgesia (PCIA): Sufentanil 2-3 mcg/kg + Flurbiprofen 200mg + Palonosetron 0.5mg (total 100 mL, background infusion 1.5mL/h, 1 ml demand dose, 15 min lockout interval)	Patient-controlled intravenous analgesia (PCIA): Esketamine 2-3 mg/kg + Flurbiprofen 200mg + Palonosetron 0.5mg (total 100 mL, background infusion 1.5mL/h, 1 ml demand dose, 15 min lockout interval)

MAC, minimum alveolar concentration; IV, intravenous; US, ultrasound.

### Cytokine and macrophages surface markers quantification by enzyme-linked immunosorbent assay and flow cytometry

We collected 10 ml of whole blood from each patient in the OFA and OA group at four time points: before anesthesia, at the end of the surgery, and on postoperative 24 hours and postoperative 48 hours.

We transferred 4 ml of whole blood into separate tubes with blood clot activating gel for serum extraction (cytokine assay). We measured the serum level of IL-10, IL-12, IL-1β, TGF-β, and TNF-α with ELISA kits (Abcam) following the manufacturer’s protocol.

We collected 6 ml of whole blood into heparin tubes for peripheral blood mononuclear cells (PBMCs) isolation using Ficoll-Paque (GE Health-care) density gradient centrifugation as standard procedure. Then we labeled PBMCs samples with FITC-conjugated anti-human CD68 (BD Biosciences) and PE-conjugated anti-human CD163 (BD Biosciences). We washed labeled cells three times in flow cytometry buffer before flow cytometric analysis on flow cytometer (Beckman–Coulter) according to the manufacturer’s instructions.

### Quality of recovery-15 questionnaire scores, visual analog scale scores, and postoperative complications

The QoR-15 is a survey consisting of 15 questions used to measure a patient’s QoR, including pain, nausea, sleep and well being. Each question is rated on a Likert scale from 0 to 10, with a maximum score of 150, indicating ideal health status (32)(**Supplementary Figure S1**). A anesthesiologist who was unaware of the group allocation evaluated QoR-15 questionnaire scores before surgery, on postoperative hours 24 and 48, respectively. And we also record postoperative pain intensity by the visual analogue scale (VAS) at 6, 24, and 48 hours after surgery. Other information was recorded from the electronic medical record including gender, age, body mass index (BMI), ASA grade, tumor and node stage, perioperative white blood cell (WBC), neutrophil, and hemoglobin, vasoactive drugs dose, hypotension, hypertension, anesthesia and surgery duration, fluid infusion, blood loss, urinary volume, extubation time, duration in PACU, time of the first flatus passage, remove drainage tube time, and some postoperative complications (nausea and vomiting, dizziness, infection).

### Statistical analysis

We performed all statistical analyses with SPSS 26.0. We reported continuous data as mean (standard deviation, SD) or median (interquartile range, IQR) based on the normality of distribution. We used the chi-squared test to compare categorical variables and presented them as number (percentage). We considered *P<*0.05 as statistically significant.

## Results

### Patient characteristics

**Figure 1:**
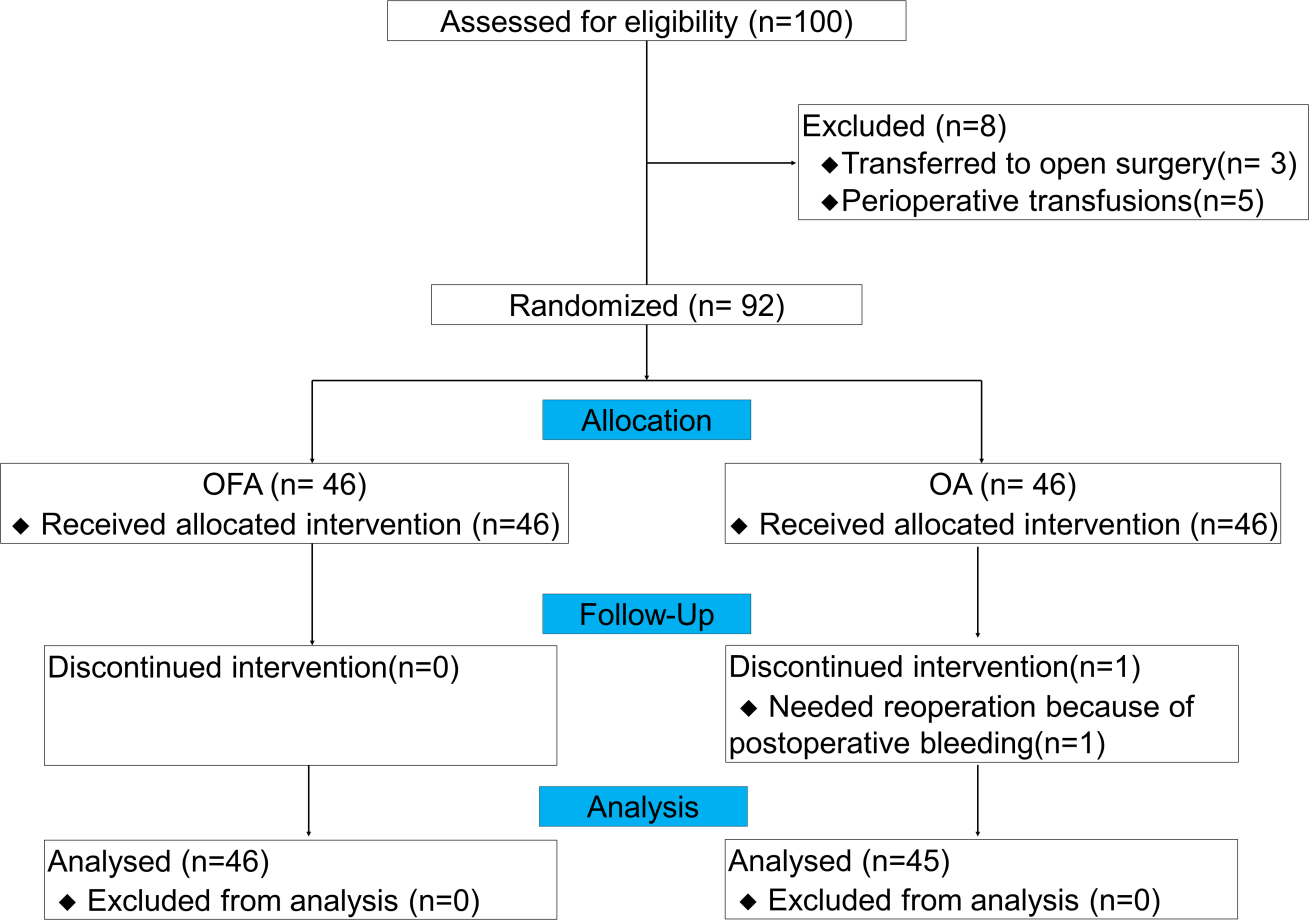
Flowchart of study subjects.

From July 2022 to December 2023, we assessed 100 patients for this study, and 92 patients were finally included (3 patients transferred to open surgery, and 5 patients with perioperative transfusions). During the follow-up period, 1 patient received reoperation due to postoperative bleeding in OA group, none of the patients were transferred to the ICU, developed respiratory failure, needed reintubation, or died. Therefore, 46 patients in the OFA group and 45 in the OA group were finally enrolled in the analysis (**Figure 1**). Among all LAGC patients in the two groups, there were no significant differences in gender, age, BMI, ASA grade, clinical tumor stage and node stage, preoperative white blood cell (WBC), preoperative neutrophil, preoperative hemoglobin, neoadjuvant therapy regimens, anesthesia duration, surgery duration, fluid infusion volumes, blood loss volume, urinary volume, drainage tube removal time, or postoperative infections (defined as any clinical-related infection after gastrectomy and before first discharge) (all *P* > 0.05) (**Table 2**).

**Table 2:** Clinical characteristics.

	OFA (n=46)	OA (n=45)	*P*-value
Gender (Male/Female)	26/20	23/22	0.676
Age (year, IQR)	59 (51.5-64)	61 (54-65)	0.230
BMI (kg/m^2^, IQR)	24 (22-25.3)	24.2 (23-25)	0.310
ASA (I/II/III)	6/34/6	4/36/5	0.649
Clinical tumor stage (cT3/cT4a)	19/27	16/29	0.668
Clinical node stage (cN1/cN2/cN3)	20/15/11	19/16/10	0.954
Preoperative WBC (10^9^/L, SD)	6.00 (2.33)	6.39 (2.46)	0.440
Preoperative neutrophil (10^9^/L, SD)	3.53 (1.59)	3.92 (1.80)	0.282
Preoperative hemoglobin (g/L, SD)	104.9 (13.3)	102.3 (12.1)	0.324
Neoadjuvant therapy (chemo/chemo+anti-PD-1)	22/24	23/22	0.459
Norepinephrine dose (mg, IQR)	0.8 (0.25-1.11)	1.05 (0.7-1.42)	0.003
Hypotension (n, %)	4	12	0.024
Hypertension (n, %)	2	1	0.570
Duration of anesthesia (min, SD)	174.9 (39.4)	170.5 (36.7)	0.579
Duration of surgery (min, SD)	132.4 (37.7)	139.1 (35.7)	0.677
Fluid infusion volume (ml, IQR)	1100 (750-1250)	1125 (800-1350)	0.319
Blood loss volume (mL, IQR)	50 (50-100)	50 (50-100)	0.963
Urinary volume (ml, IQR)	350 (300-500)	400 (350-500)	0.479
Extubation time (min, SD)	15.2 (4.9)	12.2 (4.2)	0.003
Duration in PACU (min, SD)	39.39 (9.1)	35.75 (8.0)	0.045
Nausea and vomiting (n, %)	5 (10.9%)	21 (46.7%)	< 0.001
Dizziness (n, %)	4 (8.7%)	12 (26.7%)	0.024
Time to passage of flatus (day, IQR)	3 (3-3)	3 (3-4)	< 0.001
Remove drainage tube time (day, IQR)	8 (7-9)	8 (7.5-9)	0.452
Postoperative infection (n, %)	4	2	0.414
POD1 WBC (10^9^/L, SD)	9.98 (2.60)	10.43 (2.40)	0.399
POD1 neutrophil (10^9^/L, SD)	5.65 (1.68)	6.00 (1.66)	0.315
POD1 hemoglobin (g/L, SD)	99.73 (13.11)	96.444 (11.59)	0.208

BMI, body mass index; ASA, American Society of Anesthesiologists; IQR, interquartile range; SD, standard deviation; PACU, post anesthesia care unit; POD, postoperative day; WBC, white blood cell.

The OFA group had significantly longer extubation time and PACU stay than the OA group, whereas the OA group had significantly higher rate of hypotension, higher doses of norepinephrine, higher PONV and dizziness rate, and delayed flatus passage time, compared to the OFA group (*P <* 0.05) (**Table 2**).

### QoR-15 scores and VAS scores

All patients filled out the QoR-15 questionnaire without any problems. The QoR-15 score before surgery and postoperative 48 h were not significantly different between the OFA group and OA group, *P >* 0.05, **Table 3**). However, the QoR-15 score on postoperative 24 h was significantly higher in OFA group than in OA group (114.5 [109-118.25] in OFA group vs 110 [107.5-115] in OA group, *P <* 0.05, **Table 3**). Likewise, the VAS score at 6, 24, or 48 hours postoperatively did not differ significantly between the two groups (*P >* 0.05).

**Table 3:** The QoR-15 and VAS scores.

	OFA (n=46)	OA (n=45)	*P*-value
QoR-15			
Preoperation (IQR)	130 (126-134)	128 (124.5-132.5)	0.243
At 24h (IQR)	114.5 (109-118.25)	110 (107.5-115)	0.019
At 48h (IQR)	124.5 (120-127.25)	122 (119.5-126.5)	0.093
VAS scores			
At 6h (IQR)	4 (4-5)	4 (4-5)	0.071
At 24h (IQR)	3 (3-4)	3 (3-3)	0.110
At 48h (IQR)	2 (2-2)	2 (1-2)	0.157

QoR-15, quality of recovery-15; VAS, visual analog scale; IQR, interquartile range.

### Cytokine concentrations and the ratio of macrophage subsets

**Figure 2:**
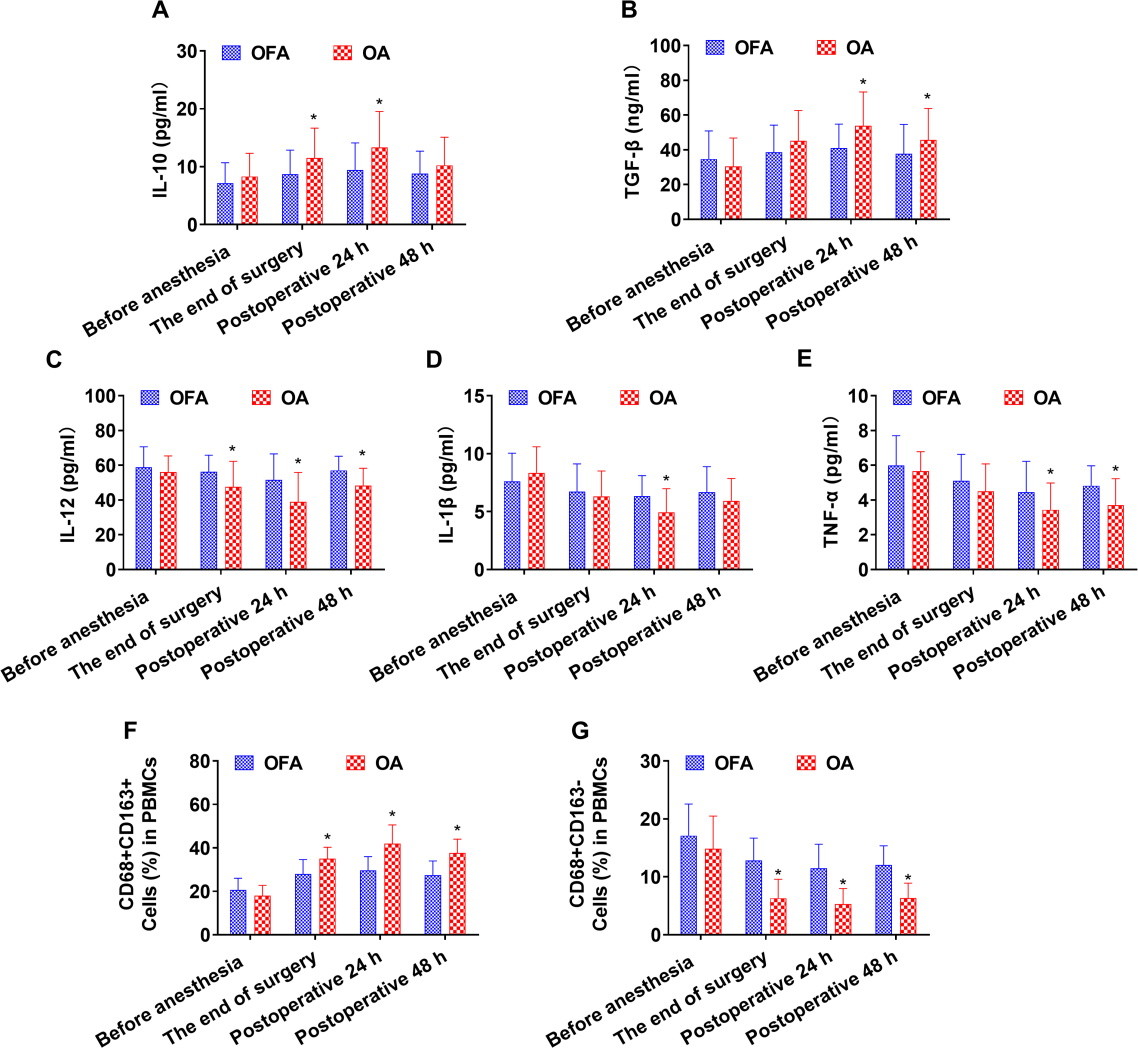
Cytokine concentrations and the ratio of macrophage subsets in total patients at different intraoperative times. **(A–E)**. Serum level of IL-10, TGF-β, IL-12, IL-1β, TNF-α measured by ELISA. **(F–G)**. Quantification analysis of CD68+CD163+ and CD68+CD163− macrophages in PBMCs. Data were shown as the mean (SD). *P < 0.05.

At the end of the surgery, the OA group had higher levels of IL-10 and CD68+CD163+ macrophages than the OFA group (*P <* 0.05). In contrast, the OA group had lower levels of IL-12 and CD68+CD163− macrophages than the OFA group (*P <* 0.05). As depicted in **Figure 2** and **Supplementary Figure S2**.

At postoperative 24 h, the OA group had higher levels of IL-10, TGF-β, and CD68+CD163+ macrophages than the OFA group (*P <* 0.05). On the other hand, the OA group had lower levels of IL-12, IL-1β, TNF-α, and CD68+CD163− macrophages than the OFA group (*P <* 0.05). As depicted in **Figure 2** and **Supplementary Figure S2**.

At postoperative 48 h, the OA group had higher levels of TGF-β and CD68+CD163+ macrophages than the OFA group (*P <* 0.05). Conversely, the OA group had lower levels of IL-12, TNF-α, and CD68+CD163− macrophages than the OFA group (*P <* 0.05). As depicted in **Figure 2** and **Supplementary Figure S2**.

### Subgroup analysis for cytokine concentrations and the ratio of macrophage subsets

We performed subgroup analysis of cytokine levels and macrophage subset ratios in LAGC patients who received neoadjuvant chemotherapy (chemo subgroup) or neoadjuvant PD-1 inhibitor + chemotherapy (PD-1 inhibitor subgroup).

**Figure 3:**
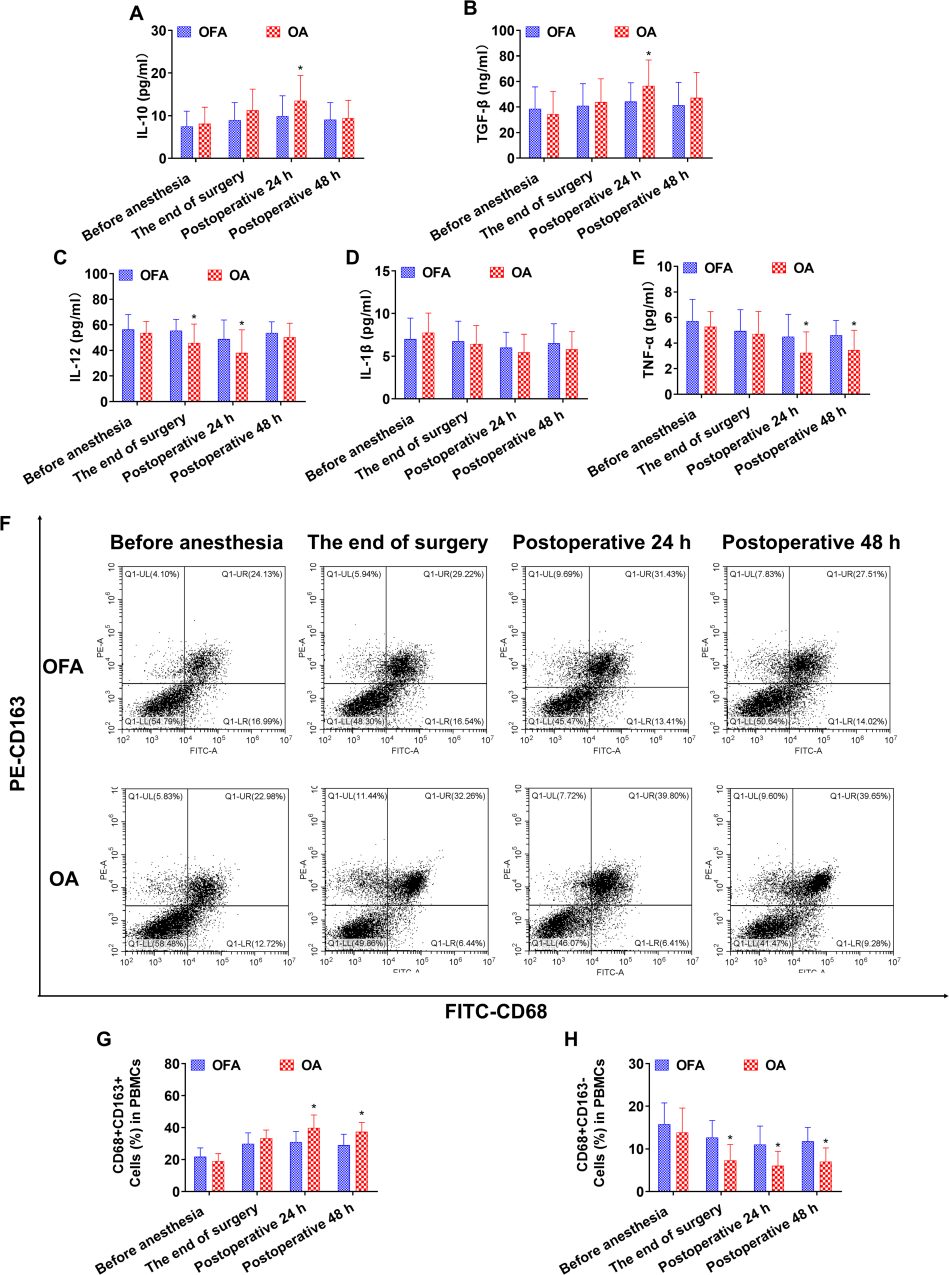
Cytokine concentrations and the ratio of macrophage subsets in chemo subgroup at different intraoperative times. **(A–E)**. Serum level of IL-10, TGF-β, IL-12, IL-1β, TNF-α measured by ELISA. **(F)**. Proportion of CD68+CD163+ and CD68+CD163− macrophages in PBMCs measured by flow cytometry. **(G, H)**. Quantification analysis of CD68+CD163+ and CD68+CD163− macrophages in PBMCs. Data were shown as the mean (SD). *P < 0.05.

**Figure 4:**
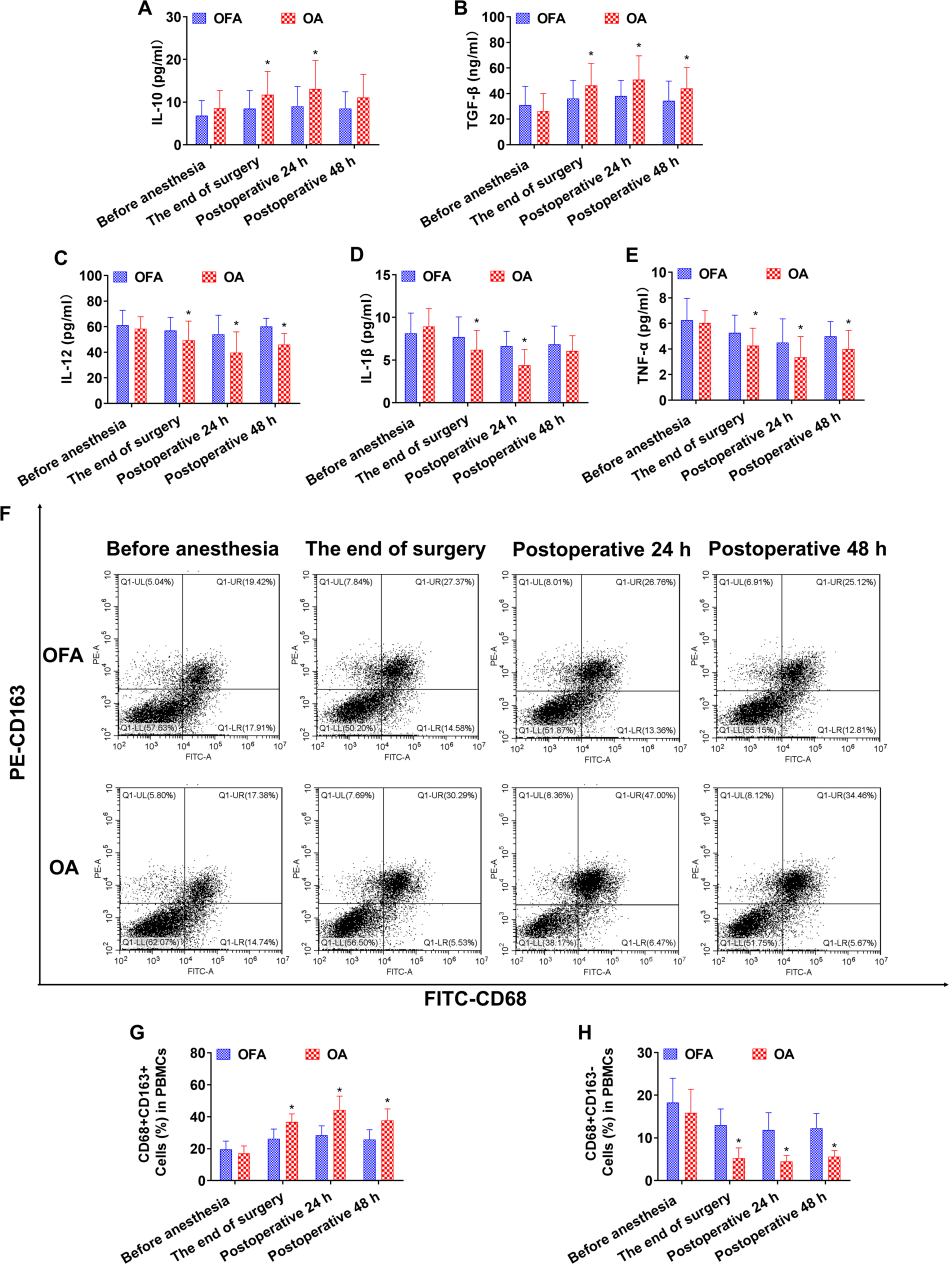
Cytokine concentrations and the ratio of macrophage subsets in PD-1 inhibitor subgroup at different intraoperative times. **(A–E)**. Serum level of IL-10, TGF-β, IL-12, IL-1β, TNF-α measured by ELISA. **(F)**. Proportion of CD68+CD163+ and CD68+CD163− macrophages in PBMCs measured by flow cytometry. **(G, H)**. Quantification analysis of CD68+CD163+ and CD68+CD163− macrophages in PBMCs. Data were shown as the mean (SD). *P < 0.05.

At the end of the surgery in the chemo subgroup, the OA group had lower levels of IL-12 and CD68+CD163− macrophages than the OFA group (*P <* 0.05) (**Figure 3**). At the end of the surgery in the PD-1 inhibitor subgroup, the IL-10 and TGF-β concentrations, and the proportion of CD68+CD163+ macrophages in the OA group were significantly higher than those in the OFA group (*P <* 0.05); the IL-12, IL-1β, and TNF-α concentrations, and the proportion of CD68+CD163− macrophages in the OA group were significantly lower than those in the OFA group (*P <* 0.05). As depicted in **Figure 4**.

At postoperative 24 h in subgroup of chemo, the IL-10 and TGF-β concentrations, and the proportion of CD68+CD163+ macrophages in the OA group were significantly higher than those in the OFA group (*P <* 0.05); the IL-12 and TNF-α concentrations, and the proportion of CD68+CD163− macrophages in the OA group were significantly lower than those in the OFA group (*P <* 0.05) (**Figures 3**). At postoperative 24 h in the PD-1 inhibitor subgroup, the IL-10 and TGF-β concentrations, and the proportion of CD68+CD163+ macrophages in the OA group were significantly higher than those in the OFA group (*P <* 0.05); the IL-12, IL-1β, and TNF-α concentrations, and the proportion of CD68+CD163− macrophages in the OA group were significantly lower than those in the OFA group (*P <* 0.05) (**Figure 4**).

At postoperative 48 h in subgroup of chemo, only the proportion of CD68+CD163+ macrophages in the OA group was significantly higher than that in the OFA group (*P <* 0.05); the TNF-α concentration and the proportion of CD68+CD163− macrophages in the OA group were significantly lower than those in the OFA group (*P <* 0.05) (**Figure 3**). At postoperative 48 h in subgroup of PD-1 inhibitor, the TGF-β concentration and the proportion of CD68+CD163+ macrophages in the OA group were significantly higher than those in the OFA group (*P <* 0.05); the IL-12 and TNF-α concentrations, and the proportion of CD68+CD163− macrophages in the OA group were significantly lower than those in the OFA group (*P <* 0.05) (**Figure 4**).

## Discussion

This prospective randomized study showed that the OFA regimen basing on continuous infusion of esketamine and dexmedetomidine improved the quality of recovery, decreases the incidence of PONV and dizziness, and reduced the time to first flatus compared with OA anesthesia in LAGC patients. However, patients receiving OFA had delayed extubation and longer stay in the PACU.

It was reported that esketamine improved the quality of postoperative recovery after surgery on postoperative days 1 and 3 in breast cancer patients (33). Other clinical studies proved that intravenous dexmedetomidine reduced PONV rate, improved quality of postoperative recovery, and alleviated the intensity of postoperative pain (34–36). In this study, we revealed higher QoR-15 scores, lower PONV and dizziness rates, and quicker first flatus in OFA groups than those in OA group. Intravenous dexmedetomidine associate with longer awaken and extubation times (37, 38), and esketamine infusion can prolong recovery time (39). Consistently, we found that the extubation time and PACU stay were longer in OFA group than those in OA group, indicating that dexmedetomidine plus esketamine resulted in better sedation.

By measuring the cytokine concentrations in serum, we found that LAGC patients in the OA group had increased levels of anti-inflammatory cytokines (IL-10 and TGF-β) and decreased levels of pro-inflammatory cytokines (IL-12, IL-1β, and TNF-α) than those in the OFA group. Consistent with the results of cytokine, LAGC patients in the OA group had higher proportion of CD68+CD163+ and lower proportion of CD68+CD163−macrophages than those in the OFA group. Subgroup analysis shows that OA caused a faster shift from M1 to M2 phenotype in the PD-1 inhibitor subgroup than that in the chemo subgroup.

In the TME, programmed death ligand-1(PD-L1) expressed on the surface of tumor cells can bind to PD-1 on T cells, which resist the killing effect of T cells and eventually cause tumor immune escape. The utilization of PD-1 inhibitor to block the PD-1/PD-L1 signal has demonstrated remarkable anti-cancer effectiveness across a diverse range of solid tumors (40). The infiltration of T cells in TME is a prerequisite for anti-tumor immunity, while the infiltration of immunosuppressive cells is a prerequisite for tumor immune escape. In the process of anti-PD-1/PD-L1 immunotherapy, M2 macrophages are immunosuppressive cells which can induce drug resistance to PD-1/PD-L1 therapy by inhibiting T-cell activity and enhancing the expression of PD-L1 in the TME (41, 42).

The immunomodulatory effects of opioids may be mediated by opioid receptors expressed in immune cells such as neutrophils, NK cells, T cells and equally in macrophages (43). And opioid receptors are the main target receptors for potent opioids such as morphine and fentanyl (44). Gong et al. investigated the effects of opioids on regulatory T cells frequencies and found that fentanyl and sufentanil could exacerbate immunosuppression via expansion of CD4+CD25+Foxp3+ regulatory T cells population *in vitro* (45). Khabbazi et al. revealed that morphine can regulate the production of macrophage proteases and M2 polarization via preventing MMP-9 increase and arginase-1 induction in TME of breast cancer, and morphine antagonists (naloxone and methylnaltrexone) can reverse this process (46).

It is also revealed that opioid receptor agonists or antagonists regulate macrophage function, thereby affecting tumor progression. Leu-enkephalin, an opioid receptor agonist, was predicted to have anti-survival effects in clear cell renal cell carcinoma (ccRCC), mainly through Th2 immunity and NRF2-dependent macrophage networks (47). Conversely, the opioid receptor antagonist, naloxone, was predicted to have pro-survival effects in ccRCC, primarily through angiogenesis, fatty acid metabolism, and hemopoiesis pathways (47). Low-dose naltrexone inhibits colorectal cancer progression and promotes tumor apoptosis by increasing M1 macrophages via the Bax/Bcl-2/caspase-3/PARP pathway (48).

Botticelli et al (49) conducted an observational, multicenter, retrospective study to explore the relationship between the administration of concomitant to ICIs (such as opioids) and the prognosis in order to evaluate a possible negative drug interaction able to impair the ICIs. Results showed that opioids use during immunotherapy is associated with early progression, potentially representing a predictive factor for PFS and negatively influencing OS as well.

Consistent with the previous researches, our study also found that the use of OA may cause more shift from M1 to M2 phenotype, and subgroup analysis shows that OA caused a faster shift from M1 to M2 phenotype in the PD-1 inhibitor subgroup than that in the chemo subgroup, suggesting that OFA may be more appropriate for LAGC patients receiving neoadjuvant PD-1 inhibitor. Our findings suggest the urgent need to further explore the impact of opioids on immune system modulation and their negative role in the response to immunotherapy treatment.

Our study had some limitations. First, we only assessed macrophage subsets and cytokines. The immune system is a complicated system. There are many other immune cells, such as NK cells, and other immune factors such as chemokines, which could be explored in the future. Second, we employed a combination of lidocaine, esketamine, and dexmedetomidine to replace opioid drugs, making it difficult to distinguish which specific drugs or methods directly affect inflammatory factors and macrophages, thereby complicating the identification of a precise causal relationship. According to this concept, other non-opioid drugs can be added arbitrarily to enhance the anesthetic effect, suppress intraoperative stress, and ensure the quality of postoperative recovery. Last but not least, we could not obtain the long-term outcomes of these patients, such as long-term survival, relapse and metastasis rates, at this time. Further studies are needed to confirm the findings here in more detail.

In summary, our results showed that OFA attenuated perioperative immunosuppression by diminishing M2 and promoting M1 macrophage polarization in LAGC patients, especially in LAGC patients treated with neoadjuvant PD-1 inhibitor.

## Data Availability

The original contributions presented in the study are included in the article/**Supplementary Material**. Further inquiries can be directed to the corresponding authors.

## Supplementary material

Supplementary Figure 1 The quality of recovery (QoR)-15 score questionnaire.

Supplementary Figure 2 Perioperative trends of cytokine concentrations and the ratio of macrophage subsets in total patients. **(A-E)**. Serum level of IL-10, TGF-β, IL-12, IL-1β, TNF-α measured by ELISA. **(F-G)**. Quantification analysis of CD68+CD163+ and CD68+CD163− macrophages in PBMCs. Data were shown as the mean (SD). **P <* 0.05 in OFA group, *#P <* 0.05 in OA group. ns, no statistical difference.
