# Supplementary figures and images for: Opioid−free anesthesia attenuates perioperative immunosuppression by regulating macrophages polarization in gastric cancer patients treated with neoadjuvant PD-1 inhibitor
Source: Front Immunol. 2024 Sep 23;15:1438859. doi: 10.3389/fimmu.2024.1438859 (PMC11488646; doi:10.3389/fimmu.2024.1438859)

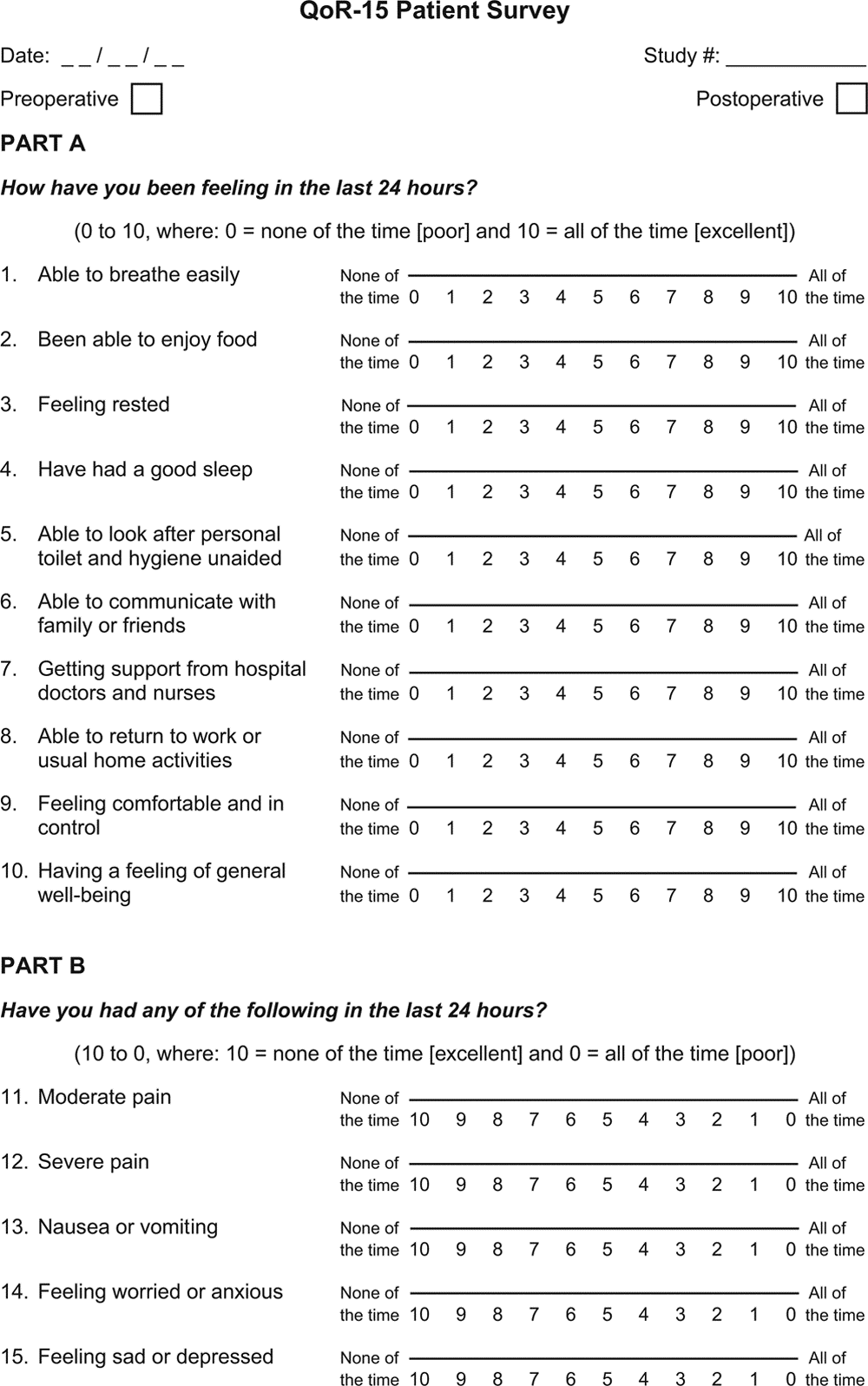

Supplement: Supplementary Figure 1 — The quality of recovery (QoR)-15 score questionnaire. [file Image1.tif]

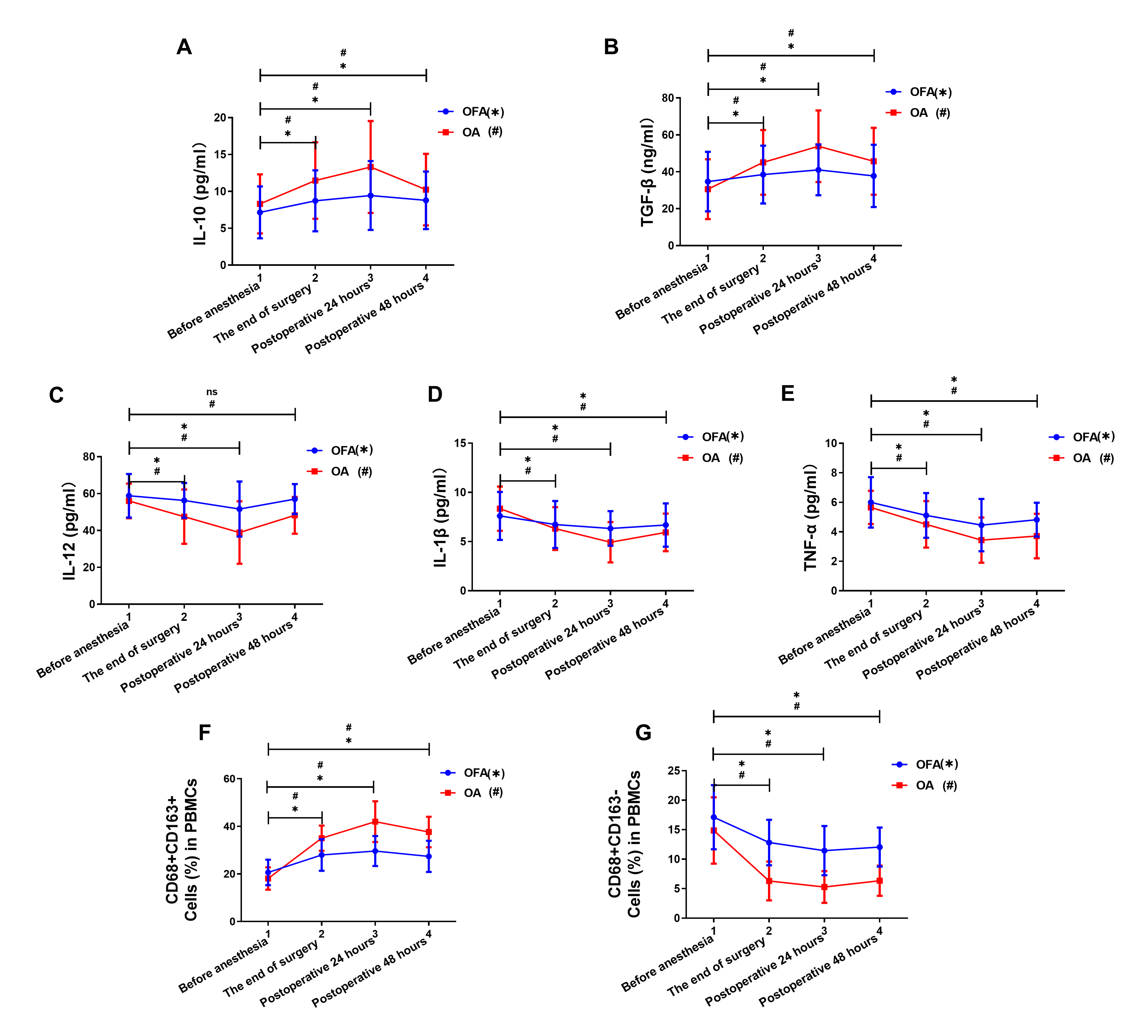

Supplement: Supplementary Figure 2 — Perioperative trends of cytokine concentrations and the ratio of macrophage subsets in total patients. (A-E). Serum level of IL-10, TGF-β, IL-12, IL-1β, TNF-α measured by ELISA. (F-G). Quantification analysis of CD68+CD163+ and CD68+CD163− macrophages in PBMCs. Data were shown as the mean (SD). *P < 0.05 in OFA group, #P < 0.05 in OA group. ns, no statistical difference. [file Image2.tif]
